# Recent developments in the engineered biosynthesis of fungal meroterpenoids

**DOI:** 10.3762/bjoc.20.50

**Published:** 2024-03-13

**Authors:** Zhiyang Quan, Takayoshi Awakawa

**Affiliations:** 1 RIKEN Center for Sustainable Resource Science, Wako, Saitama 351-0198, Japan

**Keywords:** αKG-dependent dioxygenases, enzyme engineering, fungal meroterpenoids, synthetic biology, terpene cyclases

## Abstract

Meroterpenoids are hybrid compounds that are partially derived from terpenoids. This group of natural products displays large structural diversity, and many members exhibit beneficial biological activities. This mini-review highlights recent advances in the engineered biosynthesis of meroterpenoid compounds with C15 and C20 terpenoid moieties, with the reconstruction of fungal meroterpenoid biosynthetic pathways in heterologous expression hosts and the mutagenesis of key enzymes, including terpene cyclases and α-ketoglutarate (αKG)-dependent dioxygenases, that contribute to the structural diversity. Notable progress in genome sequencing has led to the discovery of many novel genes encoding these enzymes, while continued efforts in X-ray crystallographic analyses of these enzymes and the invention of AlphaFold2 have facilitated access to their structures. Structure-based mutagenesis combined with applications of unnatural substrates has further diversified the catalytic repertoire of these enzymes. The information in this review provides useful knowledge for the design of biosynthetic machineries to produce a variety of bioactive meroterpenoids.

## Introduction

**Figure 1:**
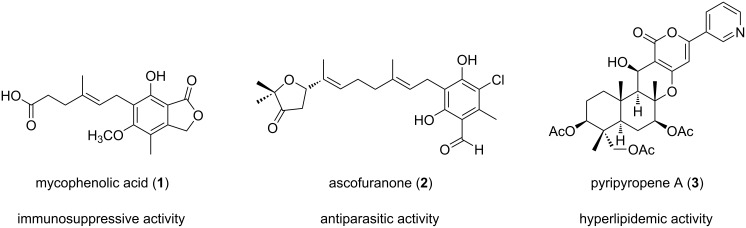
Examples of bioactive fungal meroterpenoids.

**Figure 2:**
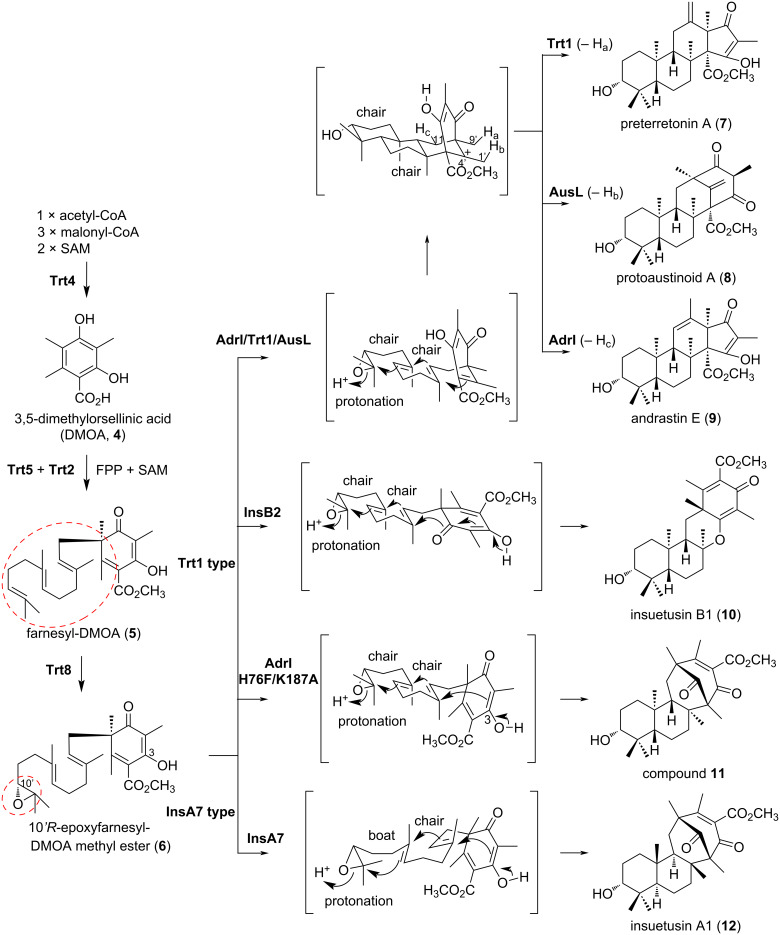
The diversity of DMOA-derived meroterpenoid biosyntheses.

**Figure 3:**
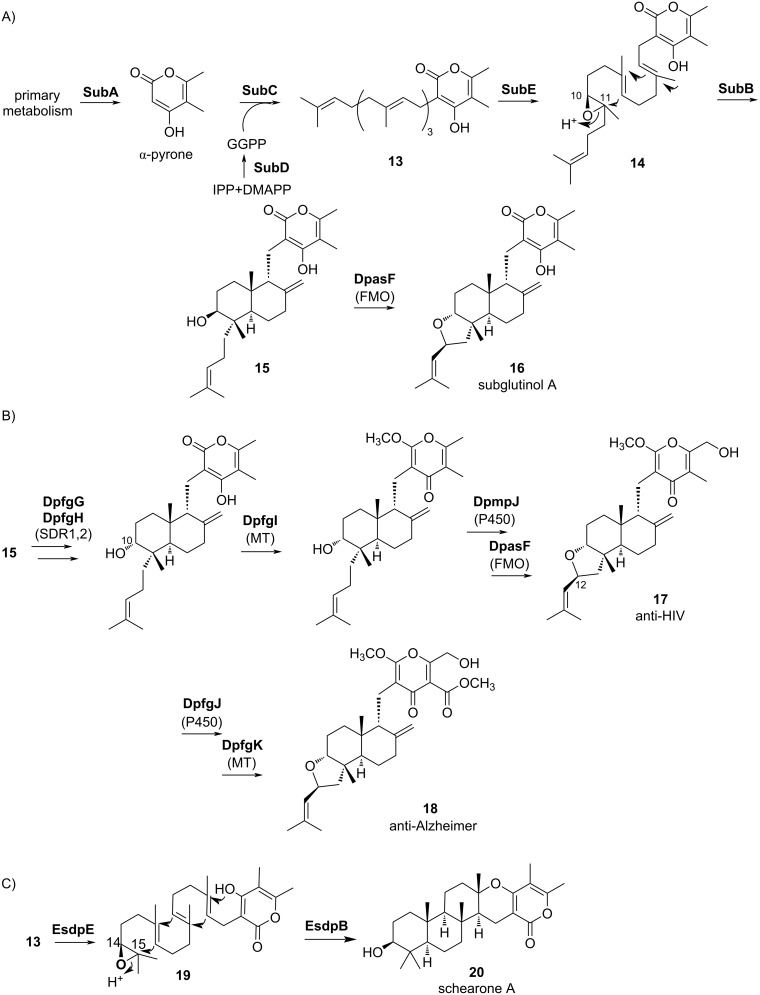
The combinatorial biosynthesis of diterpene pyrone meroterpenoids. The production of subglutinol A by SubABCDE + DpasF (A), the production of novel diterpene pyrones by SubABCDE + various tailoring enzymes (B), and the production of schearone A by SubACD + EsdpE + EsdpB (C).

Meroterpenoids are complex natural products with intricate skeletal structures, and are partially derived from terpenoids [1]. Many fungal meroterpenoids are composed of polyketide and terpenoid moieties. Examples of fungal meroterpenoids include mycophenolic acid (Figure 1, **1**), which shows immunosuppressive activity and cell differentiation-inducing activity by inhibiting the IMP dehydrogenase involved in inosinic acid metabolism [2]; ascofuranone (Figure 1, **2**), which exhibits antiparasitic activity by selectively inhibiting the oxidase of *Plasmodium trypanosoma* [3]; and pyripyropene (Figure 1, **3**), which displays hyperlipidemia treatment activity through the inhibition of acyl-CoA: cholesterol acyltransferase [4]. In this mini-review, we focus on the fungal meroterpenoids biosynthesis, especially terpenonid cyclizations and post-cyclization modifications, which mostly contribute to the skeletal diversity. Several terpenoid cyclases and αKG-dependent dioxygenases will be discussed as examples of engineering biosynthetic pathways and key enzymes involved in fungal meroterpenoid biosynthesis. Furthermore, a construction of the artificial biosynthetic pathway composed of the fungal meroterpenoids pathway and the pathway from other species, in fungal host *Aspergillus oryzae*, will be also introduced.

## Review

### Standard reactions of fungal meroterpenoid biosynthesis

The skeletal diversity within this group of compounds arises from polyketide synthesis, prenyl transfer, terpenoid cyclizations, and post-cyclization modifications [5]. In these reactions, terpenoid cyclizations and post-cyclization modifications are especially important in fungal meroterpenoid biosynthesis, contributing to the structural diversity of this class of compounds. Fungal meroterpenoid cyclases (CYCs) are seven-membrane-spanning transporter-like enzymes that regulate the conformations of cationic intermediates, leading to the terpenoid structure of each product [6–7]. Particular attention has been paid to the biosynthesis of the compounds derived from farnesyl-DMOA (**5**) composed of 3,5-dimethylorsellinic acid (DMOA, **4**) and the C15 terpenoid moiety due to their structural diversity (Figure 2).

In 2012, the biosynthesis of terretonin, a meroterpenoid derived from compound **5** and produced by the filamentous fungus *Aspergillus terreus*, was investigated [8–9] using the heterologous expression host *Aspergillus oryzae* strain NSAR1, a filamentous fungus developed by multiple mutations of metabolic genes for ease of gene transfer and high substance production capabilities [10–11].

The expression of *trt4* (polyketide synthase, PKS), *trt2* (prenyltransferase, PT), *trt5* (methyltransferase, MT), *trt8* (flavin-dependent monooxygenase, FMO), and *trt1* (meroterpenoid cyclase, CYC) in *A. oryzae* NSAR1 led to the production of preterretonin A (**7**) (Figure 2) [9]. These data indicated that Trt1 protonates the epoxide of (10'*R*)-epoxyfarnesyl-DMOA-3,5-methyl ester (**6**), leading to the cyclization of the terpenoid moiety in the chair–chair conformation, and catalyzes the deprotonation of H-9' of the resulting cation intermediate at C-4' to induce an acyl shift, forming the steroid-like structure of **7** with a 6-6-6-5 ring (Figure 2).

### Swapping terpenoid cyclases in heterologous expression systems

A search of the genome database for Trt1-homolog CYCs revealed the enzyme AusL (41% identity with Trt1) in *Aspergillus nidulans*, within the same phylogenetic clade. Expression of this enzyme in place of Trt1 resulted in the formation of the 6-6-6-6-membered ring protoaustinoid A (**8**) (Figure 2) [9]. In addition, the expression of the cyclase AdrI (38% identity with Trt1) from *Penicillium chrysogenum* produced the 6-6-6-5-membered andrastin E (**9**) (Figure 2) [8]. Like Trt1, AusL and AdrI create the common cation intermediate from **6**, but they deprotonate the cationic intermediate from H-1' and H-11, respectively [12]. The differences in the structural bases of Trt1, AusL, and AdrI are quite intriguing, in that they have similar substrate binding modes but different regional specificities.

The rapid increase in genomic information has further expanded the number of available enzyme genes, thus increasing the potential for diversifying DMOA-derived meroterpenoid biosynthesis. Recently, a similar genome mining approach led to the discovery of new CYCs, InsA7 (32% identity with Trt1) and InsB2 (40% identity with Trt1) from *Aspergillus insuetus* [13]. These enzymes catalyze the formation of the terpenoid skeleton from **6**, adopting chair–boat and chair–chair conformations to create two distinct 6-6-6-6-membered ring meroterpenoids: insuetusin A1 (**12**) and insuetusin B1 (**10**), respectively (Figure 2) [9]. Like other Trt1-type enzymes, InsB2 catalyzes the protonation of the epoxide, the formation of two six-membered rings in a chair–chair conformation, but the reaction finishes with the deprotonation of the hydroxy group at C-3 to produce compound **10**. In contrast, InsA7 commonly initiates and terminates the reaction with the protonation of the epoxide and the deprotonation of OH-3, respectively, but it produces product **12** via a boat–chair conformation. Since all of the other Trt1-like cyclases catalyze the reaction with chair–chair stereocontrol, the structure basis of InsA7 should be unique. A structural comparison of InsB2 and InsA7 will help to clarify the differences in their structural bases.

Recent advances in artifical intelligence (AI) have even made the structural modeling of membrane-bound proteins possible. In a recent study, a structural model of AdrI was constructed by AlphaFold2 [14], and its product **9** was docked into the model [15]. In the complex structure, H76 and K187 are located close to the D ring of **9**, and are expected to determine the selectivity of the product. The AdrI H76F/K187A variant was created, and produced the new compound **11**, as expected. In this mutant, deprotonation occurs from the OH-3 of the folded intermediate, and the altered cyclization mode produces **11** (Figure 2).

### Reconstitution based on the several meroterpenoid pathways in heterologous expression systems

Recently, the combinatorial biosynthesis of diterpene pyrones, derived from a pyrone skeleton and a C20 terpenoid skeleton, has been achieved by combining multiple biosynthetic pathways in a fungal heterologous expression host [16]. First, *subA* (PKS), *sub*C (PT), *subD* (GGPP synthase), *subE* (FMO), and *subB* (CYC) were expressed in *A. oryzae* to produce the intermediate **15**. Next, genes encoding a short-chain oxidoreductase (SDR), methyltransferase (MT), cytochrome P450 oxygenase (P450), and FMO from various fungi were additionally expressed in *A. oryzae* expessing *subABCDE* to produce 22 bioactive meroterpenoids, including the known antitumor compound subglutinol A (**16**) (Figure 3A and B) [17]. Among the novel compounds isolated from the production system, some exhibited intruiging pharmacological activities, such as antitumor (**16**), anti-HIV activity (**17**), and anti-Alzheimer's disease properties (**18**). Furthermore, FMO EsdpE, which epoxidizes the C-14, C-15 double bond of **13**, was employed to produce **19**, and the CYC EsdpB cyclizes in a chair–chair–chair conformation to form the 6-6-6 ring structure of **20** [18–19]. This difference in the epoxidation position represents a new point of biosynthetic diversity that will receive keen attention in the future.

### Production of pharmacologically active compounds by genetic engineering of terpenoid cyclization pathways

**Figure 4:**
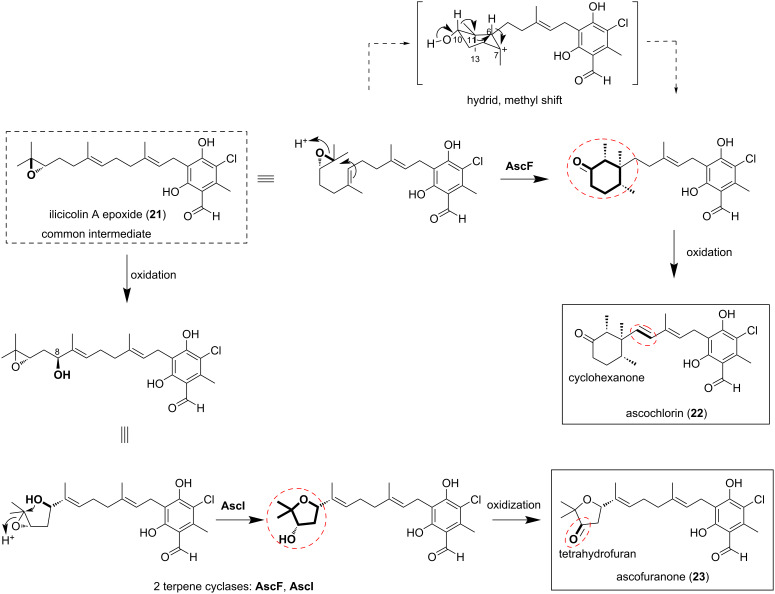
The biosynthetic reaction from the common intermediate **21** to ascochlorin (**22**) and ascofuranone (**23**), respectively.

A single filamentous fungus reportedly synthesizes two meroterpenoid compounds via two different cyclization schemes. In the filamentous fungus *Acremonium egypticum*, both asochlorin (**22**) and ascofuranone (**23**) are synthesized from a common intermediate, ilicicolin A epoxide (**21**), but the backbones of the terpenoid moiety are different (Figure 4) [20]. In ascochlorin biosynthesis, intermediate **21** is accepted by AscF to form the hexanone ring of **22**. The cyclization process is initiated by the protonation of the epoxide, leading to the formation of a cation at C-7. A cascade of migrations then occurs to form the final backbone: H-6 migrates to the cation C-7, the methyl group at C-13 shifts to C-6, H-10 migrates to C-11, and finally, deprotonation of the hydroxy group terminates the cyclization [20–21]. In ascofuranone biosynthesis, compound **21** is initially hydroxylated at C-8, and then the hydroxylated product is cyclized via epoxidation by AscI to form the tetrahydrofuran ring of **23**. The biosynthetic pathways of **22** and **23** were elucidated through heterologous expression, gene disruption, and in vitro reactions. The *ascF* gene was disrupted with the aim toward the mass-production of **23**, and a system for producing quantities greater than 500 mg/L was successfully established, thus achieving an industrial-level substance production system [20].

### αKG-dependent dioxygenases – important enzymes involved in post-cyclization modifications

After terpenoid cyclization, skeletal modifications are performed by P450 monooxygenases, α-ketoglutarate (αKG)-dependent dioxygenases, isomerases, and acyltransferases [5,22–25]. In particular, the functions of the αKG-dependent dioxygenase have been analyzed in detail due to its relatively small molecular weight and the low costs of its cofactors: αKG, ascorbic acid, and iron ions. The αKG reacts with iron and molecular oxygen to form the highly reactive Fe(IV)=O via oxidative decarboxylation. This active molecular species withdraws a hydrogen atom, and the generated radical induces various reactions such as hydroxylation, unsaturation, epoxidation, halogenation, endoperoxidation, and C–C bond reconstruction, leading to the formation of diverse chemical structures [22,26–31].

#### Structure-based mutagenesis of αKG-dependent dioxygenases

**Figure 5:**
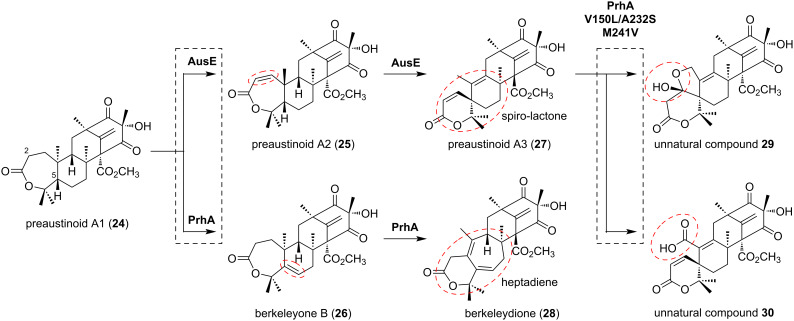
The multistep oxidations catalyzed by AusE and PrhA from the common intermediate **24**.

The first αKG-dependent dioxygenases characterized in fungal meroterpenoid biosynthesis, AusE and PrhA, share high identity (76% identity to each other) and accept the meroterpenoid preaustinoid A1 (**24**) to yield the products preaustinoid A3 (**27**) and berkeleydione (**28**), respectively (Figure 5) [32–33]. In the initial reaction, AusE abstracts H-2 of **24**, while PrhA abstracts H-5 of **24**. PrhA and AusE form a double bond to yield preaustinoid A2 (**25**) and berkeleyone B (**26**), respectively (Figure 5). AusE abstracts H-5 of **25**, forming a radical that initiates a sequential reaction to produce the spirolactone **27**. In contrast, PrhA dehydrogenates H-1 of **26** to form the heptadiene **28**. Comparing the active centers, only three amino acid residues differ between AusE and PrhA: L150(AusE)/V150(PrhA), S232(AusE)/A232(PrhA), and V241(AusE)/M241(PrhA). By swapping these residues, the catalytic activities of these enzymes were exchanged [34]. From **24**, PrhA V150L/A232S produced **27**, and AusE L150V/S232A produced **28**. Surprisingly, PrhA V150L/A232S/M241V catalyzed additional oxidations of **27** to produce compounds **29** and **30**, as unnatural products. As demonstrated in these reactions, the terpenoid skeleton undergoes significant structural changes due to radical formation through oxidase-induced hydrogen atom abstraction. Close examinations of the substrate complex structures of these αKG-dependent dioxygenases involved in these meroterpenoid oxidations revealed that the terpenoid moiety is held in a substrate pocket consisting of hydrophobic amino acids, and the polyketide moiety is retained by a loop structure serving as a lid. The conformation of the terpenoid moiety can be easily altered by mutations to the enzyme’s substrate retention site, thereby changing the position of hydrogen atom abstraction and the subsequent skeletal reorganization reaction mode, resulting in the synthesis of a variety of skeletons. Thus, the meroterpenoid αKG-dependent dioxygenase is a highly potent biosynthetic enzyme, and the structure of the product can be easily altered by mutagenesis.

#### Application of diverse unnatural substrates to αKG-dependent dioxygenases

**Figure 6:**
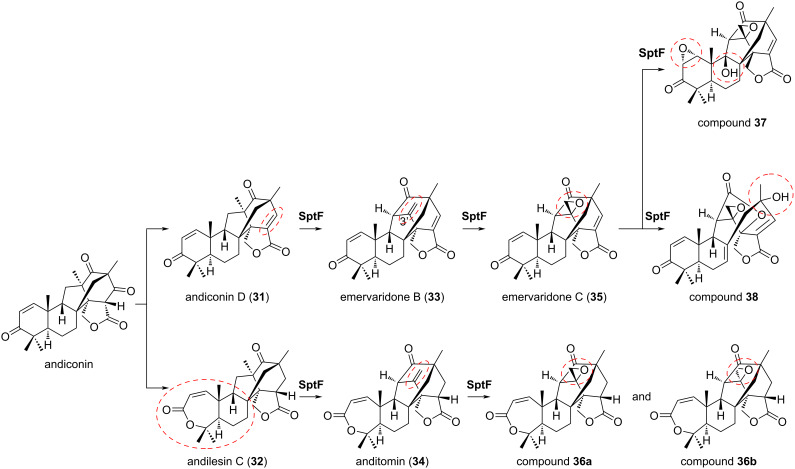
Reactions of SptF with native substrates **31** and **32**.

SptF, an α-KG dioxygenase, represents another example of structure-based engineering to create multiple products from a single enzyme reaction [35]. The natural substrates of SptF, andiconin D (**31**) and andilesin C (**32**) derived from andiconin, can be converted into emervaridone B (**33**) and anditomin (**34**), respectively (Figure 6). Subsequently, **33** can be further modified into emervaridone C (**35**), which can be oxidized to **37** or **38** (Figure 6). In contrast, **34** is converted to compounds **36a** and **36b** (Figure 6). These multistep reactions with several substrates illustrate the broad substrate specificity of SptF.

**Figure 7:**
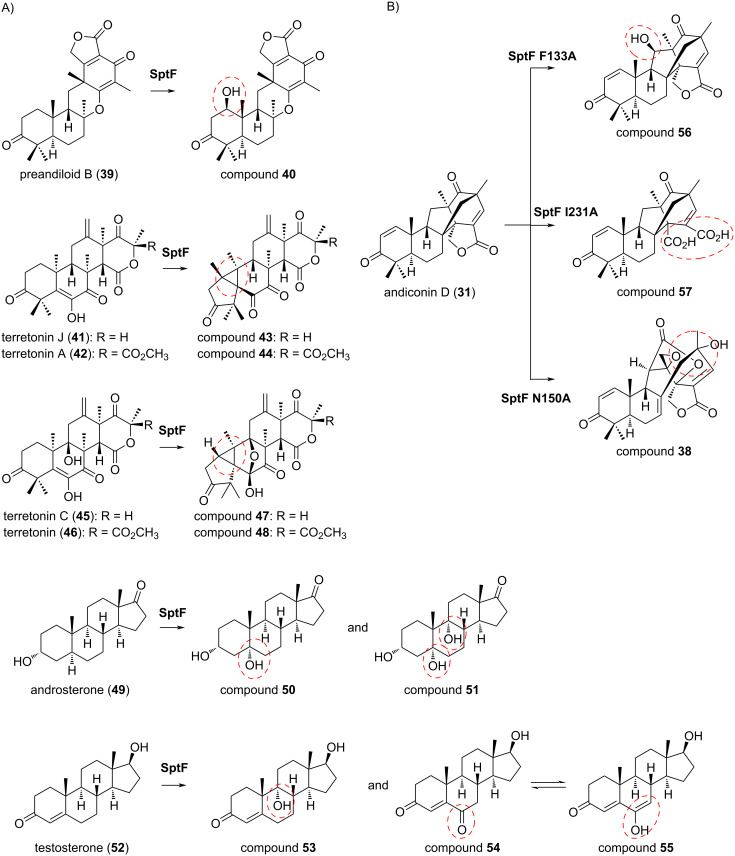
A) Reactions of SptF with unnatural substrates. B) Reactions of SptF variants with **31**.

To apply the high potential of SptF to generate a variety of oxidation products, meroterpenoids (**39**, **41**, **42**, **45**, and **46**) and terpenoids (**49** and **52**) were also used as unnatural substrates to construct a series of new compounds (**40**, **43**, **44**, **47**, **48**, **50**, **51**, and **53**–**55**) (Figure 7A). In addition, a structure-based mutagenesis study of SptF was performed to further amplify its catalytic potential. Firstly, the hydrophobic residues Ile63, Phe133, and Ile231, which compose the substrate binding site of SptF, were mutated. As a result, SptF F133A oxidized **31** into **56**, while I231A oxidized the same substrate **31** into compound **57** (Figure 7B). Then, the hydrophilic residues Asn65, Ser114, Thr148, and Asn150, which also line the cavity, were also mutated. Compound **31** was converted into **38** by SptF N150A, while the WT enzyme converted **31** into **37** and **38** (Figure 7B). These results exemplify the production of oxidized terpenoids and meroterpenoids through the use of non-natural substrates and the application of structure-based mutagenesis.

#### Evolution of αKG-dependent dioxygenase with saturated mutagenesis

**Figure 8:**
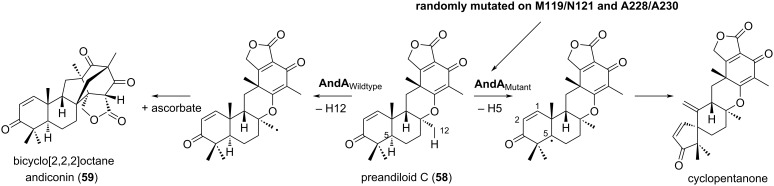
The reaction of the αKG enzyme AndA and its variants generated via saturated mutagenesis.

As meroterpenoids readily change their reaction products depending on the conformation of the terpenoid skeleton, the regiospecificity of the oxidation reaction can be modified by introducing random mutations in the substrate-binding site of αKG-dependent dioxygenase. The αKG-dependent dioxygenase AndA withdraws H-12 of preandiloid C (**58**), and generates a radical leading to the construction of the bicyclo[2.2.2]octane skeleton of andiconin (**59**) (Figure 8) [36–37]. Two amino acid pairs, M119/N121 and A228/A230, in its substrate-binding pocket were mutated by saturation mutagenesis using degenerate primers, resulting in an enzyme that oxidizes C-5, thereby reacting with the electron between C-1 and C-2 [38]. In the future, such mutagenesis strategies will be applied to a wider range of oxidases to alter their reactivities and create an expanded range of products.

#### Construction of artificial fungi–plant hybrid biosynthetic pathways

**Figure 9:**
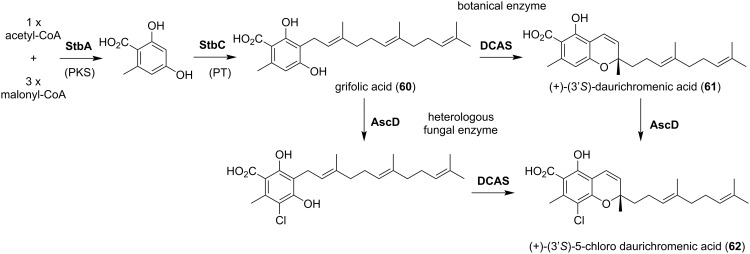
The synthetic biological production of daurichromenic acid and its halogenated derivative.

Furthermore, there are examples of the enzymatic synthesis of plant-derived pharmaceutical meroterpenoids through the heterologous expression of a combination of fungal and plant biosynthetic enzymes. The biosynthetic enzymes StbA (polyketide synthase, PKS) and StbC (prenyltransferase, PT) from the filamentous fungus *Stachbotrys bisbyi* were produced in *A. oryzae* to synthesize grifolic acid (**60**) (Figure 9) [39]. In addition, the gene encoding DCAS, a prenyl group oxidase from the plant *Rhododendron dauricum*, was additionally expressed in *A. oryzae* expressing *stbAC* after codon-optimization to produce daurichromenic acid (**61**) (Figure 9), a plant-derived meroterpenoid that exhibits anti-HIV activity [40]. Furthermore, by expressing AscD, a fungal halogenase from the ascochlorin biosynthetic pathway, the authors succeeded in biosynthesizing **62**, an unnatural meroterpneoid, chloro-daurichromenic acid, halogenated at C-3 (Figure 9). Today, with the improvement of gene synthesis and expression technologies, genes from a wide variety of species are available, further increasing the potential for applications of biogenetic resources. In the future, advances in synthetic biology will enable the biosynthesis of bioactive compounds with still greater skeletal diversity.

## Conclusion

In summary, many novel terpene cyclases and αKG-dependent dioxygenases were discovered by recent developments in genome mining approaches. Unique meroterpenoids have been generated by integrating these enzymes into meroterpenoid biosynthetic pathways, enzymatic engineering, and feeding with unnatural substrates. Enzyme crystal structure analysis and AI structure prediction also facilitated these investigations. Besides terpene cyclases and αKG-dependent dioxygenases, P450 monooxygenases and UbiA-type prenyltransferases have also expanded the variety of meroterpenoids [41–42]. These untapped membrane enzymes will be applicable for engineered biosynthesis, with structural models providing useful information for mutagenesis, as in the engineering of meroterpenoid cyclases [11] and fungal P450 oxygenases catalyzing cross-coupling between two aromatic rings [43]. In the future, further developments in bioinformatics, structural biology, and AI techniques will enable the design of biosynthetic enzymes and pathways to produce desired bioactive compounds, although understanding the chemistry catalyzed by individual enzymes will remain important. Furthermore, highly productive substance production systems will be developed through host, metabolome, and enzyme improvements by genome editing technology.

## Data Availability

Data sharing is not applicable as no new data was generated or analyzed in this study.
